# Identifying Learner Profiles Through Universal Screening: Academic Anxiety and Depression in Nepalese University Students

**DOI:** 10.3390/bs16040557

**Published:** 2026-04-08

**Authors:** Dev Bandhu Poudel, Jerrell C. Cassady, C. Addison Helsper

**Affiliations:** 1 Department of Humanities and Social Sciences, G.P. Koirala Memorial Community College, Kathmandu 44602, Bagmati, Nepal; 2 Department of Humanities and Social Sciences, Brooklyn College, Kathmandu 44600, Bagmati, Nepal; 3 Research Department, Sambhavya Foundation, Kathmandu 44600, Bagmati, Nepal; 4 Research Design Studio, Ball State University, Muncie, IN 47306, USA; cahelsper@gmail.com

**Keywords:** academic anxiety, test anxiety, scale validation, cultural comparison, student depression

## Abstract

As in other cultures, university students in Nepal struggle with significant academic pressure, which often leads to academic anxiety and depression. The current study aims to expand awareness of the presence, prevalence, and impact of student academic anxiety and depression among Nepalese university students as well as to test an emerging approach to universal screening to identify learners’ need profiles to promote targeted intervention supports. Participants included 547 Nepalese college students who completed the Academic Anxiety Scale (AAS) and the University Student Depression Inventory (USDI). Confirmatory factor analysis (CFA) was conducted to evaluate the validity of the Nepalese versions. Finally, comparative analyses using an archival dataset of students from the United States explored consistencies across cultural contexts. Nepalese translations of both scales demonstrated high reliability and validity and identified similarities in patterns of expressed academic anxiety and depression across cultures. Furthermore, four profiles of need were generated based on levels of anxiety, depression, and academic motivation. The results supported clear recommendations for tiered interventions in specific domains of emotion regulation. This initial large-scale study of academic anxiety and depression in a Nepalese university population provided confirmation that the models of anxiety and depression as well as incidence levels were consistent with existing research from other contexts. Moreover, the results provided strong confirmation that universal screening with simplified self-report measures can identify clear patterns of need among students, which can be aligned with targeted tiered interventions to support student thriving.

## 1. Introduction

Estimates widely vary based on the measures of anxiety and depression employed, but research has repeatedly demonstrated that between 20 and 45% of students are likely to report debilitating anxiety or depression at some point over the course of their academic careers, and that the probability increases progressively as students move through the grade levels—with the highest rates occurring in university students (Greenbaum et al., 1999; Kessler et al., 2005). Similarly, longitudinal studies have provided reliable evidence that students experiencing anxiety or depression tend to have lower performance, lower life satisfaction, and less success in achieving positive employment outcomes (Bullis & Cheney, 1999; Carter & Wehby, 2003; Wood & Cronin, 1999). For university settings, anxiety and depression indicators are both linked to lower rates of retention and program completion.

In many Western industrialized countries, access to higher education has accelerated in the past two decades, resulting in a more diverse student body. The diversification of university students has led to increased rates of first-generation and low-income students, who routinely are found to have lower levels of “academic readiness” (Musawar & Zulfiqar, 2025). One of the notable influences on the increase in access in many countries has been a shift away from the “entrance exams” that were traditionally used to screen or filter learners who were expected to struggle in university settings (College Board, 2025). Many educational reformists advocated for the removal of entrance exams to reduce assessment biases that often favored economically privileged learners. However, we argue that as university populations continue to shift in their readiness profiles, the university must similarly adapt. As such, we have been working to draw attention to the power of universal assessment strategies that focus on learners’ skill profiles—not to restrict access to a university, but to identify the supports that are most likely needed to ensure they make the transition to a new academic community. This study is part of an ongoing international initiative to test collections of student profile assessment tools that can be used by university leaders to ensure that targeted tiered intervention services can be directed toward learners with identified needs. These strategies focus on “push-in” support systems (e.g., Gonçalves et al., 2025) to proactively serve learners at risk of university struggles. The primary shift in this approach is to step away from standard university support systems that are predominantly “self-selecting” and implement an identification and alert system that supports levels of intervention that are commensurate with the needs demonstrated by learners in university settings.

### 1.1. University Student Academic Anxiety

Anxiety in academic contexts is noted as a global concern. PISA studies have demonstrated that roughly 60% of 15-year-olds worldwide report that test anxiety or math anxiety present barriers to optimal performance (OECD, 2017, 2023). The research has also demonstrated that the level of reported anxiety related to academic stressors increases across the academic timeline, reaching a peak incidence level during university (Cassady et al., 2019). In addition to the increased demands based on the rigor expected as students progress into university settings, university students often face these heightened challenges without the educational, social, and family supports that have supported their ability to cope with academic challenges in the past (Vredenburg et al., 1988).

The experience of anxiety deriving from a multitude of varying academic challenges is termed academic anxiety and encompasses various domains of academic-specific anxieties such as test anxiety, reading anxiety, and math anxiety (Cassady, 2010). Models explaining the formation of anxiety for academic events generally converge on students’ appraisals of their ability to successfully manage the challenge (or threat) imposed by a task or setting (e.g., Lazarus, 2006). In these models, the first appraisal determines the degree of challenge likely imposed in the event, but the critical point that indicates the probability of anxiety arises when the learner estimates the resources and abilities at their disposal (skills, abilities, support network) to successfully meet those expectations (Cassady, 2022).

Manifestations of academic anxiety can involve the fear of underperforming compared to one’s peers, concerns about managing responsibilities effectively, or the experience of stress within classroom settings (Cassady, 2010, 2022). Academic anxieties have been routinely identified as corresponding with suboptimal levels of academic performance as well as with indicators of social, emotional, and psychological well-being for students across all age ranges (Cassady, 2022; Levine, 2008). It is linked to procrastination in older adolescents and perceived barriers, lower achievement, and less effective resource management in late-starting female students (Milgram & Toubiana, 1999; Cassady & Johnson, 2002; Heller & Cassady, 2017). Additionally, anxiety negatively impacts student retention and success and diminishes students’ efforts and motivation, affecting their overall academic performance (S. K. Das et al., 2014).

### 1.2. University Student Depression

In academic settings, Lee et al. (2021) identified depression as a significant burden in the United States, with a higher prevalence (36% moderate to severe) among undergraduate students. Similarly, in college students, the prevalence of depression was reported at 39.88%, with 22.42% experiencing mild depression, 13.69% moderate depression, and 3.77% severe depression (Khadka et al., 2022). University students exhibit elevated rates of depression compared to the prevalence observed in the general population (Eisenberg et al., 2007; Mellin, 2008; Ibrahim et al., 2012; Lim et al., 2018).

Depressive issues and related symptoms impact students’ physical, emotional, cognitive, and interpersonal abilities (Kitzrow, 2003). Depressive symptoms correlate with impairments in information processing (Lyubomirsky et al., 2003), time management, problem solving and decision making (I. Das & Mishra, 2010). Additionally, it adversely affects academic performance (Kitzrow, 2003). Depressive symptoms in university students are also negatively associated with perceived connectedness to the university and positively correlated with perceived stress and concern over mistakes and doubts about actions, academic stress, maladaptive perfectionism and low levels of problem-focused coping (Khawaja et al., 2021).

There has been increased focus on the unique nature of university students’ manifestations of depressive symptoms as compared to the standard population’s clinical depression symptomology (Coyne, 1994; Steer & Clark, 1997). In particular, fluctuating patterns of sleep and appetite among university students are more likely due to lifestyle and environmental factors associated with university life, rather than to depressive issues (Coyne, 1994; Steer & Clark, 1997). One classic study on student depression indicated that the level of severity was relatively mild but chronic, suggesting a pattern of ongoing yet not clinically identified depressive symptomology (Vredenburg et al., 1988). Based on these patterns of difference in the manifestation of depression among university populations, Khawaja and Bryden (2006) developed the University Student Depression Inventory (USDI), a 30-item scale measuring depressive symptomology across three subscales: Lethargy, Cognitive/Emotional, and Academic Motivation. Their work has demonstrated that the scale is effective at differentiating levels of depression indicators for students in university settings in ways that are more contextually relevant than more traditional measures that do not focus on the unique conditions facing learners in higher education (Khawaja & Bryden, 2006; Romaniuk & Khawaja, 2013).

### 1.3. Universal Assessment to Promote Thriving

The traditional approach to student support in university settings rested largely on relying on individual students to be self-advocates, where they identify their needs and access the resources that support those needs. While most universities have some form of a student support network, research repeatedly demonstrates that students at risk of dropping out of higher education are less likely to know about or to access the support systems that are available. To remediate that pattern, we have proposed intentional universal assessment programs in university settings to identify profiles of learners that can then prompt outreach by student support professionals to increase the level of engagement they have with strategies that should be directly linked to their needs. The core underlying theoretical perspective of universal assessment rests on the presumption that services that are activated only after student failure in higher education are generally “too little, too late.” Universal assessment strategies help detect needs early and can be used to guide learners to intervention, support networks, or social groups that can encourage skill development, engagement, and eventual success (Glover & Albers, 2007; Bruhn et al., 2014).

Data captured in universal assessment programs not only identify learners who would benefit from developing skills in emotion regulation or self-regulated learning strategies, but they can also be used to identify the level of intervention support that is likely to support thriving (Marquez et al., 2013). Building on these identifications of “severity level,” a multi-tiered intervention framework can be implemented, consistent with “response-to-intervention” programs (see Brown-Chidsey & Steege, 2010; Walker et al., 1996). These approaches generate a service model designed to provide intensive support for the neediest learners and have gradations of lesser support and engagement as students demonstrate fewer deficits. In the standard 3-tier models, the expectation is that Tier 1 interventions are provided to all learners—generally providing core information or general strategies that promote academic success (Horner et al., 2010; Seeley et al., 2010). Tier 2 interventions are focused on students who demonstrate an inability to thrive with just exposure to Tier 1 supports or who demonstrate characteristics that align with likely difficulty or failure. Tier 2 engages learners in more intensive training and support (e.g., small group sessions, coaching). Finally, in the classic 3-Tier model, those students who demonstrate continued difficulty despite supports in the Tier 1 and Tier 2 interventions are referred for additional support (e.g., 1-1 interventions, clinical support). Pairing universal assessment with a tiered intervention approach allows students to be “filtered” into levels of need without having to wait for students to experience failures in the academic setting.

### 1.4. Current Study

The current study was created in response to the growing evidence that identifying and supporting students who experience elevated academic anxiety and/or depressive symptomology is an effective strategy to promote student retention and success (e.g., Serrano Pintado & Escolar Llamazares, 2014). While much of this work has been conducted in North and South America, Western European, and select Asian countries (e.g., China, Singapore, and India), there is growing awareness of the need to expand the research base to be more representative of the global population. To date, there have been no identified published studies providing a validated measure of academic anxiety or depressive symptomology in a Nepalese university population. Nepal’s classification as a developing country points to clear differences in access to higher education, but a few pointed details are important for context. Nepal’s average literacy level is 66% (with some groups reporting a level as low as 46%; Neupane, 2020). International data tracking sources (e.g., UNESCO) identify that just under 50% of students complete “upper secondary” programs (which require completion of an entrance exam), and approximately 21% of the Nepalese population attends post-secondary institutions. Collectively, compared to university students in more established and wealthy countries, it is expected that students in Nepal experience fewer educational supports leading to post-secondary programs and are more likely to experience financial challenges to continuing their education (UNESCO Institute for Statistics, 2026). While we acknowledge the heightened barriers that the average student in Nepal is likely to encounter, our focus is on the individual psychological challenges and how those translate to student experience in their own contexts. In essence, our study intends to directly explore the level of anxiety and depression indicators reported by university students in Nepal to determine if the existing research has any bearing on their experience, with the long-term goal of working toward systems of identifying student need profiles that can reasonably be adapted across cultural settings (UNESCO Institute for Statistics, 2026).

This study first established the construct validity of Nepalese versions of both the Academic Anxiety Scale (AAS) and the University Student Depression Inventory (USDI). Second, the relationships among the measures were evaluated to identify the level of concordance of the Nepalese sample with international trends. Third, specific cross-cultural comparisons on the academic anxiety measure were conducted to examine consistency and divergence between Nepal and the US. Finally, we used these data to conduct a test of the utility of a profiling system to identify profiles of students with depression and anxiety. By doing so, we hoped to validate a reliable set of measures that could serve as a needs profile assessment model targeting university student learner profiles.

## 2. Materials and Methods

### 2.1. Sample

The study included a sample of 547 Nepalese university students, ranging in age from 18 to 49 (M = 21.46, SD = 3.70), recruited from multiple colleges across the Kathmandu Valley using an opportunity-based stratified sampling method to ensure representation across three university classifications (Community College, Government College, and Private College). As Kathmandu serves as an educational hub, the sample included students from diverse regions and backgrounds. Information on the demographic characteristics of participants was solicited from a range of variables, including age, major, gender, ethnicity, academic condition, and living location (see Table 1). For these variables, participants were given the option to opt out of responding as part of their consent process. As such, our use of pairwise deletion (shown in Table 1) results in variations in total N for each variable, but the reported values are based on the number of responses for that variable. Conversely, the students were unable to submit the survey without complete responses to the primary measures of interest (Academic Anxiety Scale and University Student Depression Inventory).

Data were collected in the Fall Semester of 2023 after permission to contact students to solicit voluntary participation in this study was secured from each participating institution. Students were contacted by email, where they received a consent form including details on their right to withdraw from participation and a link to contact the first author with questions before proceeding to the online survey.

### 2.2. Measures

The academic anxiety and student depression measures were both translated into Nepali following a multi-phase translation process focused on transliteral equivalence and use of culturally appropriate terminology. First, each scale was translated into Nepali by a native speaker who was a language expert with an academic background in English. That version was subsequently edited by another native Nepali teacher to ensure natural linguistic flow and ecological validity for native speakers. In addition, the items were reviewed by a Nepali psychology lecturer to ensure clarity, appropriateness, and fidelity to psychological constructs. The refined Nepali versions were subsequently back-translated into English by an English teacher who was not familiar with the original surveys. These back-translated versions (along with the original English versions) were distributed to five experts who were fluent English speakers to comment on any variations from the original and translated versions. No suggestions for correction or adaptation were noted in this final step.

Academic Anxiety. The Academic Anxiety Scale (AAS) was developed by Cassady et al. (2019) and captures various manifestations of anxiety experienced by learners in educational environments (see Appendix A). The scale includes 11 items that employ a four-point Likert-type response format (1—“not at all typical of me,” 2 = “somewhat typical of me,” 3 = “quite typical of me,” and 4 = “very typical of me”; see Cassady, 2022). Cronbach’s alpha for the AAS was reported to be 0.87, indicating high levels of internal consistency reliability (Heller & Cassady, 2017). Furthermore, students’ scores on the AAS showed significant moderate-to-strong correlations with the Beck Depression Inventory (BDI-II) as well as measures of neuroticism and cognitive test anxiety (Cassady et al., 2019).

In a recent analysis, severity standards for the AAS were established with a large University sample using Latent Class Analysis to test the viability of empirically derived “levels” of reported academic anxiety. The response patterns indicated four discernible levels of academic anxiety, which were identified as Non-Anxious, Mild Academic Anxiety, Moderate Academic Anxiety, and High Academic Anxiety (Finch et al., 2024). These categorical groupings provide a simplified method for identifying a level of need for support, but these specific levels have not been validated in a non-US cultural context. For the purposes of examining cross-cultural consistency in item responses on the AAS, item-level response data from the Finch et al. (2024) study were secured (N = 1266, 69% female, 86% White).

Depressive Symptoms. The University Student Depression Inventory (USDI) is a 30-item self-report questionnaire originally developed for use with Australian university students that utilizes a 5-point Likert-type scale (1 = “not at all,” 2 = “rarely,” 3 = “sometimes,” 4 = “often,” and 5 = “all the time”; Khawaja & Bryden, 2006). The scale has three subscales—Lethargy (9 items), Cognitive–Emotional (14 items) and Academic Motivation (7 items)—which have been confirmed through confirmatory factor analyses (Romaniuk & Khawaja, 2013). For all three subscales, higher levels indicate higher levels of depressive tendency. As such, “Academic Motivation” on this measure is based on items that illustrate “demotivation” (lack of interest, unlikely to engage in study behaviors). The USDI total score exhibited high internal consistency (α = 0.95), as did each of the three subscales: Lethargy (α = 0.89), Cognitive/Emotional (α = 0.92), and Academic Motivation (α = 0.84). Strong test–retest reliability was also demonstrated for the total scale (r = 0.86) and for the subscales Lethargy (r = 0.76), Cognitive/Emotional (r = 0.91), and Academic Motivation (r = 0.80) (Khawaja & Bryden, 2006). Finally, the USDI total score and subscales demonstrated internal consistency across three cross-cultural samples (i.e., Australia, Portugal, and Iran), with coefficient alpha values ranging from 0.82 to 0.95 (Khawaja et al., 2013). A Persian translation of the scale demonstrated similar qualities to the original version (Habibi et al., 2014), and the scale has also been reviewed and adjusted based on Rasch measurement explorations to identify misfitting or gender-biased items (Balbuena et al., 2021).

Convergent validity was supported by significant correlations with the Depression, Anxiety, and Stress Scales (DASS), particularly with the DASS Depression Scale (r = 0.76). Divergent validity was established through an independent sample *t*-test, showing significantly lower life satisfaction among students reporting high levels of depression on the USDI (Khawaja & Bryden, 2006). The study also proposed cut-off points for total score interpretation, enhancing the scale’s utility (Romaniuk & Khawaja, 2013).

Student Demographics. Finally, respondents gave information on a range of demographic variables relevant to university students, including residence status information, age, gender, religion, and academic indicators. The demographic data reported in this study (see Table 1) are used primarily to define the sampling frame given the noted unique characteristics of the sample from Nepal. However, comparative analyses were conducted based on gender as well as “university type” to explore if there were similarities or differences in the presentation of depressive or anxious symptoms among learners from Government, Community, or Private Colleges.

### 2.3. Data Analysis

Data were analyzed using SPSS Version 27 and Mplus version 7.31. Confirmatory factor analysis (CFA) was conducted to examine the factor structure of the AAS and USDI in the Nepalese university student population. Model fit was evaluated based on recommended values by Kline (2023): Comparative Fit Index (CFI) and Tucker–Lewis Index (TLI) greater than 0.90, Root Mean Square Error of Approximation (RMSEA) of around 0.05, and Standardized Root Mean Square Residual (SRMR) less than 0.1. For both CFAs, we employed the diagonally weighted least squares (WLSMV) estimation method, which has been validated as most appropriate for the nature of our data and sample (Li, 2016; Rhemtulla et al., 2012).

Item analyses were employed to explore the cross-cultural applicability and validation of the AAS among university student populations in Nepal and the United States (no direct access to USDI data were available for a comparable analysis). This involved comparative analyses (e.g., item means, variances, and cross-cultural differences using independent samples *t*-tests) for all items for the two samples. This approach was aimed at identifying any cultural variations on individual items for the AAS. We also compared the distribution of previously published AAS severity cut-off scores across the two cultural settings.

Finally, Latent Profile Analysis was undertaken to explore potential student profiles within the Nepalese sample based on anxiety and depression indicators. This was achieved using the R tidyLPA package, reviewing 1 through 5 classes for optimal fit. The variables used for establishing classes included the standardized values (z-scores) for AAS and the three subscales of the USDI. The final determination of optimal fit was based on a collective review of theoretical interpretation of the contributing variables and fit indicators, including SABIC, BIC, entropy, log-likelihood, and the probability estimates (prob-max) in the main profile for each participant (Ferguson et al., 2019). Comparisons among the defined profiles were used to explore variations among the established groups, with a focus on meaningful differentiation of experiences with anxiety and depression.

## 3. Results

### 3.1. Validating the Factor Structure of Nepalese AAS

Confirmatory factor analysis was conducted to test the validity of the Nepalese translation of the AAS. Overall, the measure demonstrated a reasonable fit to the proposed single-factor model. The Chi-Square Test of Model Fit (χ^2^ = 159.88, df = 44, *p* < 0.001) indicated a significant difference between the model and the observed data. However, the chi-square statistic is sensitive to sample size and should be interpreted cautiously as it generally overestimates model misfit for large samples (Meade & Lautenschlager, 2004). The CFI (0.956) and TLI (0.945) both surpassed the threshold of 0.90, indicating a good fit of the model to the observed data. The RMSEA (0.069) and SRMR (0.051) were both slightly above the ideal thresholds but continued to support a reasonable model fit.

Review of factor loadings for the 11 items of the Nepalese version of the AAS demonstrated that they were all statistically significant (*p* < 0.001), indicating that each item meaningfully contributed to the latent construct of academic anxiety. Furthermore, standardized loadings ranged from 0.456 to 0.765, reflecting a strong relationship between each item and the underlying single-factor construct of academic anxiety (see Table 2).

### 3.2. Reliability of Nepalese AAS

For our reliability analysis, we adopted a dual approach to reliability estimation, encompassing both traditional (Cronbach’s alpha) and more nuanced methods tailored for ordinal data. Given the ordinal nature of AAS item responses, we also calculated a reliability coefficient using polychoric correlation matrices. This method is particularly suited for ordered categorical data and is argued to yield more accurate estimates of internal consistency (Gadermann et al., 2019). Both approaches yielded strong internal consistency; Cronbach’s alpha for the full 11-item scale was 0.82, and the polychoric reliability coefficient further substantiated this finding (α = 0.88, 95% CI = 0.72–0.96).

Finally, as shown in Table 2, individual item “raw alpha” values are provided, which demonstrate the contribution of each item to the overall Cronbach’s alpha value. Examination of these items generally focuses on looking for items that are diverging from the other items (indicating an ill-fitting item in building the total scale value). As indicated, the items performed in a uniform fashion.

### 3.3. AAS Cross-Cultural Comparisons

**Figure 1:**
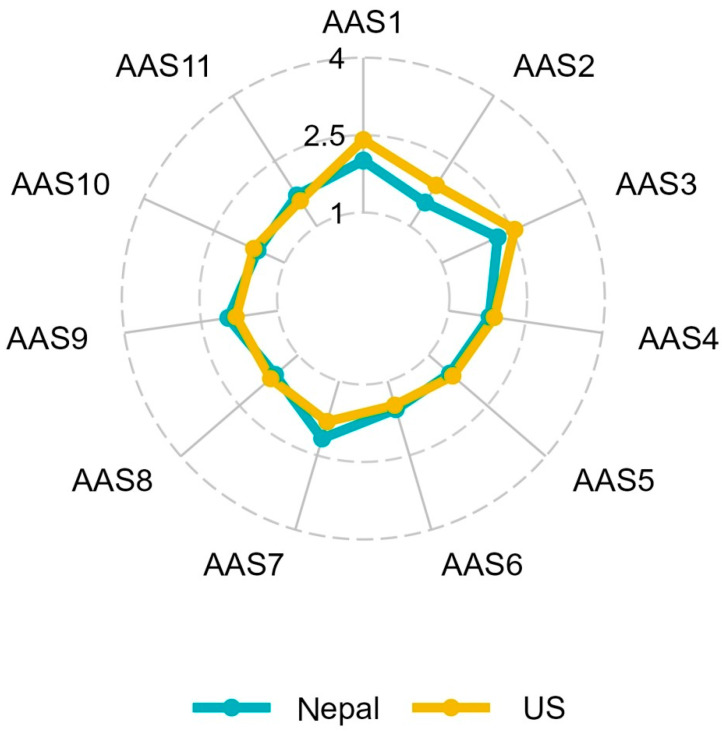
Radar chart of AAS item comparison across Nepalese and US samples.

Comparison of student responses to the AAS across the US and Nepal involved examining items for differential means and variances as well as comparing the mean values of each item for differences (independent *t*-tests; see Table 1). Comparisons across items demonstrated that US students rated the first three items higher than the students in Nepal. Those items address heightened worry that “my best is not good enough” and that they are “not doing assignments properly,” as well as a tendency to procrastinate. One item was rated higher by Nepalese learners, identifying that they spend more time at school “worrying about what’s next.” Despite these differences, the overall pattern indicates relative consistency in the items that were likely to be rated highest or lowest across the two samples (see Table 2 and Figure 1).

Our analysis focused on the distribution of students on the severity levels of the AAS, which revealed that the Non-Anxious and High Anxiety groups were reasonably similar in the percentage of students across the two countries (see Table 3). However, there was noted divergence in the distribution of students in the Mild and Moderate levels of academic anxiety, with roughly a 10% difference observed.

While the Nepalese sample was younger overall, both samples demonstrated a trend where the lowest levels of reported anxiety were observed for the older students in the samples. Gender distribution for the two samples differed, with the US sample having a higher proportion of females than males and the Nepalese sample having a reasonably even distribution of males and females. Examining the distribution of gender representation points to a notable difference in the relationship between gender and academic anxiety across the two cultures. That is, in the US sample there was a stronger tendency to see males reporting lower levels of AAS (note the pct male dropping over the four levels of AAS). Such a trend was not observed for the Nepalese sample, which indicated a relatively similar gender distribution across all four levels of reported academic anxiety.

### 3.4. USDI Validity and Reliability

The structural validity of the USDI was first examined using a one-factor model to act as the base comparison to the empirically supported three-factor model containing Cognitive–Emotional (CE), Lethargy (LE), and Academic Motivation (AM). CFA model fit results showed a moderate fit; the model yielded a chi-square value of 1684.640 with 405 degrees of freedom, indicating a significant difference from the observed data (*p* < 0.0001). The RMSEA estimate was 0.049 with a 90% confidence interval between 0.045 and 0.053, which is right on the preferred threshold of 0.05. The CFI (0.918) and TLI (0.912) both met the threshold criterion of 0.90. SRMR (0.050) indicated a poor model fit. Overall, the one-factor model displayed only moderate fit indices, suggesting that the USDI might be better represented by a more complex model.

Subsequently, we assessed the empirically supported three-factor model of the USDI, which comprises factors labeled Cognitive–Emotional (CE), Lethargy (LE), and Academic Motivation (AM). The fit indices for this model were as follows: The model yielded a chi-square value of 763.295 with 402 degrees of freedom, indicating a significant difference from the observed data (*p* < 0.0001). The RMSEA estimate improved to 0.041 with a 90% confidence interval between 0.037 and 0.046. The CFI and TLI were 0.943 and 0.938, respectively, passing the ideal threshold of 0.90. More importantly, the SRMR value decreased to 0.044, indicating a better fit as compared to the one-factor model. The factor loadings for all items in the three-factor model were statistically significant (*p* < 0.0001), demonstrating strong associations with their respective latent constructs.

Inter-factor correlations among LE, CE, and AM were all significant (*rs =* 0.82–0.87), suggesting a meaningful relationship among these dimensions of student depression. The item-specific R-square values from the confirmatory factor analysis of the University Student Depression Inventory (USDI) three-factor model indicate that the factors explained between 34.2% and 74.0% of the variance in individual items, demonstrating a substantial explanatory power of the model for each item (as shown in Table 4).

Comparatively, the three-factor model of the USDI exhibited better fit indices than the one-factor model, suggesting that the Cognitive–Emotional, Lethargy, and Academic Motivation dimensions provide a more accurate representation of the data. The significant improvement in RMSEA, CFI, TLI, and SRMR in the three-factor model indicates its superiority in capturing the complexities of depression symptoms among university students. These results provide strong support for the structural validity of the USDI in its three-factor form. However, the range of R-square values for item contribution to the USDI three-factor model indicated that differential item effectiveness was evident, suggesting that some items are more effective in capturing the latent constructs. This observation supports prior suggestions that item refinement may be viable for the USDI, trimming less influential items for more efficient assessment (see Balbuena et al., 2021).

Once again, we ran both Cronbach’s alpha and polychoric reliability estimates. For the individual factors, the Cronbach’s alphas were as follows: Lethargy (α = 0.88), Cognitive-Emotion (α = 0.93), and Academic Motivation (α = 0.85). These values suggest a high level of internal consistency within each factor. The polychoric correlation-based reliability coefficients were also strong: Lethargy factor (α = 0.90, 95% CI: 0.76–0.97), Cognitive-Emotion (α = 0.95, 95% CI: 0.89–0.98), and Academic Motivation (α = 0.89, 95% CI: 0.68–0.98). These reliability indices collectively underscore the high degree of internal consistency across both the overall scale and its individual factors.

### 3.5. AAS and USDI Convergent Validities

To evaluate the convergent validity, we conducted a correlational analysis with the three distinct factors of the USDI: Cognitive–Emotional, Lethargy, and Academic Motivation. The findings revealed a strong and significant positive correlation between the AAS and each of the USDI factors, as well as durable negative relationships among the anxiety and depression measures when examining student-reported GPA (see Table 5).

### 3.6. Latent Profiles

To examine the potential to identify groups of student needs using self-report data from university students, a Latent Profile Analysis was conducted. Our profile approach relied on only the students’ values for academic anxiety and the three subscales of depressive symptoms on the USDI. Analyses were conducted with the tidyLPA package (Rosenberg et al., 2018) in R using mclust for model estimation (Scrucca et al., 2016), examining the model fit for 1 through 5 classes.

Relying on guidelines for determining model fitness (e.g., Ferguson et al., 2019), the 4-class solution provided the best overall explanation for the sample distribution (see Table 6). While most solutions met expectations for good fit, the final decision was driven by our interpretation that adding the fourth profile resulted in a meaningful improvement in SABIC, BIC, entropy, and log-likelihood. However, the level of improvement on the measures was not as substantial. More importantly, the review of the class composition for the four-profile solution was more theoretically aligned. Review of the identified classes across each model indicated that the primary benefit of the 4-class solution was in the finer degree of clarity in estimating the “high emotion regulation” group. While this group is smaller than the others (N = 48), it provided information on a specific class of students who exhibited comparably very low levels of anxiety and depression. Small groups are to be viewed with caution in LPA, but the group is both theoretically meaningful and captures more than the recommended 5% of the sample (Ferguson et al., 2019).

Final placements for the classes reported in Table 7 were determined using the simplified one-step method of identifying the highest probability estimation for class placement generated in tidylpa with mclust. While this is the least rigorous method for determining final “group placement,” our high levels of entropy (0.855) suggest that our classification is durable.

To verify the utility of the class assignment method selected, we compared the results of the simple classification group assignment for profile membership with the more robust BCH three-step approach using the class probability estimations to account for classification uncertainty (Bolck et al., 2004). Specifically, we tested for profile group differences on GPA. Results using the weighted values were identical to the simple method when identifying group differences on GPA, *F*(3, 392) = 2.67, *p* = 0.047. Post hoc analyses for both approaches demonstrated that the difference between class 2 (T1-ER) and class 4 (T2-ER) was the only statistically significant (albeit weak) difference.

**Figure 2:**
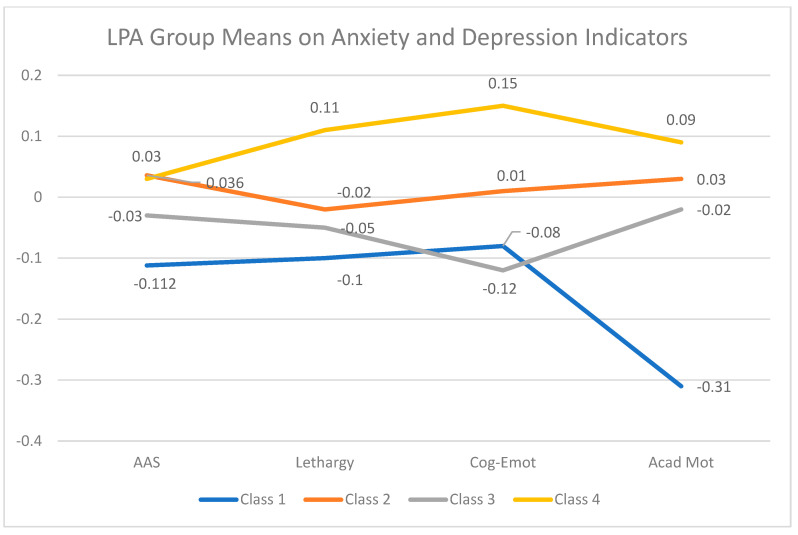
LPA group comparisons.

The LPA demonstrated four profiles representing variations in reported anxiety and depression. Mean-centered values (z-scores) for each of the four indicator variables were used to allow more reasonable comparisons of the different constructs. As such, the data displayed in Table 6 and Figure 2 indicate the relative levels of anxiety or depression for each profile. Class 1 (N = 48) had the lowest levels of reported academic anxiety as well as of depression indicators. As a reminder, the Academic Motivation subscale of the USDI is an indication of the “absence of academic motivation” such that low scores indicate high levels of academic motivation. Therefore, we consider Class 1 as capturing students with comparably high emotion regulation skills and academic motivation tendencies. We refer to this theoretically “ideal” profile for university students as “High Emotion Regulation.”

The “next best” profile regarding emotion regulation was Class 3, which demonstrated lower levels of anxiety and depression than the remaining two groups. They are primarily distinguishable from Class 1 in that they have higher levels of Academic Anxiety and report lower levels of motivation for academics. In the parlance of our approach to universal assessment to identify learners with needs, we refer to this group as “Tier 1: Motivational Support.” This profile definition is intended to recommend a level of need (Tier 1; useful, but not essential) as well as specific area(s) of need (promoting achievement motivation).

We refer to Class 2 as “Tier 1: Emotion Regulation Support.” Similar to Class 3, this group of students reports average levels of anxiety and depression indicators—but all domains are elevated beyond those of Class 1 and 3. We propose this profile would not present as “at risk,” but would experience a degree of anxiety and depression that may be considered “normal” in a college student population. As such, Tier 1 supports that direct students toward supports and resources, positive “self-care” strategies, and methods to help reduce their levels of anxiety or depression symptoms are anticipated to be sufficient for this profile.

Finally, we refer to Class 4 as “Tier 2: Emotion Regulation Support.” The classification of this group as Tier 2 is prompted by the elevated values on all domains of maladaptive student functioning focused on anxiety and depression. In tiered intervention models, this level prompts offering more direct and targeted intervention supports. Tier 2 supports may involve regular engagement in small-group settings, regular “check-ins” with paraprofessionals, or providing timely outreach to learners at critical points of likely stress (e.g., midterms, final exams, course selection for future semesters).

Review of the composition of the four classes identified indicates that the profiles did not vary across the types of academic institutions (Community-Based, Government, Private; Χ 2(6) = 6.02, *p* = 0.42; see Table 6). A review of student demographics demonstrated no significant variations based on self-reported child abuse, substance use, or risky sexual behavior. While there were no statistically significant differences based on gender distribution across the four profiles (Χ 2(3) = 6.27, *p* = 0.09), there was a noticeably higher number of females in the “High Emotion Regulation” profile and a slightly higher representation of males in the “Tier 2—Emotion Regulation” profile.

## 4. Discussion

Our study makes several contributions to the literature focused on the presence of anxiety and depression in university students. First, this study has validated the applicability of the AAS and USDI in Nepal. Second, the results of this first large-scale study in Nepal demonstrated that university student experiences largely converge with prior studies on academic anxiety and depression in other contexts. Finally, the results confirm the potential to identify typologies of student needs in emotion regulation skills using accessible and cross-culturally relevant measures. This approach to identifying student needs is in service of providing university leaders with actionable data that can be used to provide targeted support services to bolster success.

### 4.1. Validation of Nepalese AAS and USDI Assessment

The results of this study provide confirmation that the AAS and USDI measures were relevant and produced consistent values for a Nepalese population. Moreover, the relationships among the subscales of the USDI and AAS were strongly correlated with each other and were negatively related to academic performance, consistent with prior research in the US (Cassady et al., 2019). The distribution of “levels” of academic anxiety was also consistent with prior work (Finch et al., 2024). This provides evidence that the experiences of university students in countries at differing levels of educational and economic resources follow similar patterns. We find this to be encouraging evidence that systemic identification and intervention strategies may be applicable across cultural settings.

Direct comparison of item-level differences between students in Nepal and the United States illustrated significant differences in mean values for specific items. However, the pattern of item values indicated that there was reasonable construct consistency across the two populations. That is, items that received higher average response values in Nepal tended to receive similarly high relative values in the US sample. Naturally, differential manifestations of anxiety across societies are anticipated (e.g., Baghei & Cassady, 2014; Cassady et al., 2004; Furlan et al., 2009; Grabau et al., 2024; Lewis-Fernández et al., 2011). Explanations of those differences are generally supported by models of anxiety that recognize individual students’ appraisals of academic stressors or threats and their perceived coping strategies and resources to meet those threats (e.g., Pekrun, 2006; Zeidner & Matthews, 2005). Specifically, those appraisals are influenced by cultural norms (e.g., individualism vs. collectivism; Hofmann & Hinton, 2014).

While item-level variations across cultures were observed, the relative consistency in overall rates of reported Academic Anxiety in Nepal and the United States builds upon growing evidence that students identify academic stressors in university settings that they recognize as barriers to optimal functioning (Serrano Pintado & Escolar Llamazares, 2014), but the specific stressors of importance may vary based on the degree of “threat” as interpreted through their unique cultural conditions (e.g., Cassady et al., 2004; Furlan et al., 2009).

The clear distinction of three factors on the USDI, coupled with the significant correlations among those subscales (Cognitive–Emotional, Lethargy, Academic Motivation), supports the multidimensional view of university student depression offered by Khawaja and Bryden (2006). However, we must reassert that the measure is focused on the unique nature of depressive symptomology for university students—not clinical depression. As such, like academic anxiety, these data provide insight into pre-clinical indicators that may provide insight into early warning symptoms or identify students who may need additional attention to determine if there is a clinical need beyond the Tier 1 and Tier 2 recommendations we offer below for student profile usage.

### 4.2. Universal Screening of Anxiety and Depression for University Students

Functionally, the results of this study demonstrate the potential utility of universal screening measures to identify profiles of learner need in post-secondary education (see Cassady & Thomas, 2020). Specifically, our results demonstrate the viability of readily accessible pre-clinical self-report measures that can identify student emotion regulation strengths and needs in academic settings. We propose that the AAS and USDI (or similar measures) can provide academic institutions with actionable information that can be captured with minimal effort to provide early alerts for learners who are at risk of experiencing the negative academic effects associated with anxiety and depression. The importance of this universal screening process rests on bridging gaps that typically exist between students in need and the services in place. That is, most universities provide support structures for students (e.g., counseling centers, academic advisors, faculty or staff support personnel). However, the standard model for accessing supports relies on students to self-identify and initiate contact with the supports. Using universal screening measures (such as USDI and AAS) enables academic leaders and support systems to directly identify individual students who can be targeted for intervention supports.

While we believe this study provides a reasonable “proof of concept” for universal screening, the model employed in our current study is merely a start in our estimation. In particular, we advocate for adding a measure of self-regulated learning skills (e.g., effective study strategies, planning, scheduling) to build a more complete representation of student needs. While the profiling system demonstrated in this study identifies the potential to classify students based on differential emotion regulation needs as well as “attitudes toward education” (as seen in the Academic Motivation subscale in the USDI), having a specific measure of the students’ habits and skills focused on how (or if) they engage with academic tasks would provide more guidance on intervention supports (e.g., tutoring, skills training) that would promote thriving.

### 4.3. Implications for Practice

Environmental stressors such as instructors’ comments about forthcoming evaluations, social pressures from peers or family, or expectations of negative consequences for failing to meet expectations are all well-established sources of potential stressors or challenges (e.g., von der Embse & Witmer, 2014). Research has routinely demonstrated that students who experience elevated anxiety and depression symptoms have higher rates of academic failure through underperformance or withdrawal. These declines in performance are generally explained by limitations in processing efficiency or attentional control under periods of elevated stress (e.g., Eysenck et al., 2007). Similarly, students managing emotionally taxing academic conditions experience elevations in cognitive load, hampering optimal performance (Chen & Chang, 2009).

We propose that universities can more effectively mitigate negative outcomes associated with anxiety and depression by proactively identifying needs and providing directed outreach and support tailored to the students’ specific forms and levels of need (Cassady et al., 2022). Optimally, early identification and support would reduce the number of students who experience significant failures by ensuring that they build skills and strategies to manage anxiety or depression symptoms before they become overwhelming.

Basic strategies to support those learners’ needs have been offered in the literature, with growing evidence of efficacy. For instance, effective Tier 1 strategies include promoting a calm or secure environment in classrooms (Hughes & Coplan, 2018) or avoiding threat appraisals when presenting forthcoming evaluations (Segool et al., 2013). For those learners identified as needing Tier 2 supports, cognitive restructuring and relaxation techniques have been successful in tempering negative outcomes (Serrano Pintado & Escolar Llamazares, 2014). Finally, a note on Tier 3 (intense support) is important. Our goals in proposing a universal assessment process are to identify learners with needs and to serve those with Tier 1 and Tier 2 needs with emotion regulation and self-related learning skills training directly through non-clinical support mechanisms. Learners who present with extreme needs and would likely be referred to clinical support staff or to counseling may well be identified by a universal screening measure, but the referral would be to receive more direct and supported outreach from a professional clinician in those cases.

### 4.4. Limitations and Future Research

As mentioned, future research should continue to explore the presence, prevalence and consistency of student pre-clinical academic anxieties and depression in universities in diverse populations. Without clear evidence from diverse settings, we limit the long-term goal to building generalized models of emotional challenges and solutions for learners in academic settings. However, more specific recommendations for next steps include exploration of the nuanced relationships between academic anxiety and depression in a time-series analysis, which would enable the identification of a “triggering effect” such that heightened academic anxiety levels may spark depressive symptoms (or vice versa). More aligned with the goal of our proposed universal screening and tiered interventions, longitudinal studies measuring the efficacy of referring and serving students based on their needs profiles are essential. Finally, we advocate for the inclusion of self-regulated learning skill measures to augment the battery of measures currently studied. Our work is currently focused on adding that element to our profiling process to advance the holistic approach to student academic thriving in university settings.

We acknowledge that the study has limitations that affect the generalizability and precision of some analyses. For instance, this study of one developing country cannot be directly applied to all contexts. However, using such a divergent population from the US and Australia (where the primary measures were developed) and finding consistent construct validity is encouraging. Furthermore, our use of the simplified placement of participants into classes (based on the LPA) without using the more rigorous three-step models may limit the overall accuracy of the underlying latent profiles. Once again, this was a calculated decision, based on the conclusion that the end result was more ecologically consistent with the procedures that are likely to be used in university settings where this operation may be implemented. Our use of BCH-weighted estimations in the GPA analyses demonstrated that in this particular sample, our decision to use simplified methods for profile assignment was reasonable, but systemic approaches should be mindful of this distinction in the analytic use of profiles (Bolck et al., 2004).

## Figures and Tables

**Table 1:** Demographic composition.

	Frequency	Valid Pct.
**Gender (N = 539)**		
Female	278	51.58
Male	261	48.42
**Ethnicity (N = 541)**		
Brahmin/Kshetri	307	56.75
Janajaati	50	9.24
Newar	42	7.76
Rai	44	8.13
Tamang	42	7.76
Others	56	10.35
**Area of Permanent Residence (N = 491)**		
City area (urban)	300	61.10
Village Area (rural)	191	38.90
**Living Companions (N = 511)**		
Friends	34	6.65
Parents	283	55.38
Single	51	9.98
With other family member	143	27.98
**Regularity in Passing Exams**		
Kept Confidential (Chose not to reply)	61	11.58
‘Passed Regularly without Back-paper’ ^a^	324	61.48
‘Passed Despite Back-paper’	107	20.30
‘Trouble Passing the Exams’	35	6.64
**Field of Study (N = 527)**		
Business/management/administration	101	21.35
Computer Science	45	9.51
Education	38	8.03
Humanities or Social Sciences	91	19.24
Medicine, Nursing and other Health Sciences	53	11.21
Psychology	76	16.07
Science including Agriculture, Forestry and Engineering	69	14.59
**Types of Academic Institutions (N = 518)**		
Community College	97	18.73
Government College	180	34.75
Private College	241	46.53
**Years in Bachelor’s Degree (N = 230)**		
First Year	76	33.04
Second Year	75	32.61
Third Year	44	19.13
Fourth Year	35	15.22

Note: All percents reported are based on the total number of students responding to each demographic question (see category label for valid number of respondents in that category). ^a^: “Back-Paper” is a resubmission process required in test completion when performance does not meet expectations.

**Table 2:** Item statistics, factor loadings and cultural comparisons of AAS.

Nepalese (N = 547)						US (N = 1266)			
AAS Item Analysis				CFA		AAS Item Analysis			Difference
	M	SD	Raw Alpha	Loading	R^2^	M	SD	Raw Alpha	*p*
AAS1	2.008	0.874	0.90	0.456	0.792	2.408	1.051	0.82	<0.000
AAS2	1.546	0.659	0.90	0.552	0.696	1.941	0.939	0.81	<0.000
AAS3	2.188	1.096	0.89	0.491	0.759	2.552	0.887	0.81	<0.000
AAS4	1.801	0.897	0.89	0.524	0.725	1.892	0.929	0.81	0.122
AAS5	1.550	0.845	0.89	0.765	0.415	1.617	0.759	0.80	0.214
AAS6	1.571	0.854	0.89	0.735	0.46	1.492	0.599	0.80	0.105
AAS7	2.163	1.137	0.89	0.604	0.635	1.820	0.925	0.81	<0.000
AAS8	1.592	0.930	0.89	0.764	0.417	1.700	0.856	0.80	0.071
AAS9	1.973	1.041	0.89	0.661	0.563	1.824	0.942	0.80	0.014
AAS10	1.579	0.750	0.89	0.67	0.551	1.658	0.863	0.81	0.139
AAS11	1.712	0.822	0.89	0.642	0.588	1.590	0.614	0.80	0.017

Note. Mean difference *p*-values presented in the final column compare Nepal and US sample scores of individual items.

**Table 3:** AAS Severity levels—Nepal and USA.

	Mean AAS	SD AAS	N/%	Age (M)	Pct Male
Nepal					
Non-Anxious	12.3	1.20	119 (21.8%)	22.8	50.00%
Mild Anxiety	17.8	2.02	212 (38.8%)	21.7	46.40%
Mod Anxiety	25.1	2.36	177 (32.3%)	21.5	49.70%
High Anxiety	34	2.80	39 (7.1%)	21.0	51.40%
USA					
Non-Anxious	12.6	1.13	271 (20.3%)	24.1	38.40%
Mild Anxiety	17.1	1.64	444 (30.5%)	24.3	29.80%
Mod Anxiety	24.3	2.44	393 (40.2%)	21.7	24.50%
High Anxiety	34.1	3.96	139 (8.8%)	22	18.80%

Note: Ranges for levels of AA based on values identified in Finch et al. (2024). Total AAS scores for the groups are: Non-Anxious = 11–14; Mild = 15–20; Moderate = 21–29; High = 30–44.

**Table 4:** Standardized factor loadings for USDI.

Item	LE	CE	AM	R-Square
USDI1	0.652			0.576
USDI3	0.588			0.654
USDI4	0.625			0.609
USDI8	0.729			0.468
USDI10	0.777			0.396
USDI24	0.8			0.359
USDI27	0.811			0.342
USDI29	0.757			0.427
USDI30	0.714			0.491
USDI2		0.739		0.453
USDI5		0.732		0.465
USDI6		0.674		0.546
USDI9		0.652		0.575
USDI11		0.723		0.477
USDI14		0.699		0.511
USDI18		0.776		0.398
USDI20		0.86		0.26
USDI21		0.853		0.273
USDI22		0.743		0.448
USDI23		0.772		0.404
USDI25		0.829		0.313
USDI26		0.803		0.355
USDI28		0.677		0.542
USDI7			0.618	0.618
USDI12			0.704	0.504
USDI13			0.615	0.622
USDI15			0.795	0.367
USDI16			0.795	0.367
USDI17			0.761	0.421
USDI19			0.808	0.348

**Table 5:** Correlational analyses—AAS and USDI.

	AAS	Lethargy	Cog-Emot	Acad Mot	USDI-Tot
AAS					
Lethargy	0.621 ***				
Cog-Emot	0.652 ***	0.868 ***			
Acad Mot	0.587 ***	0.743 ***	0.721 ***		
USDI Total	0.674 ***	0.943 ***	0.963 ***	0.851 ***	
GPA	−0.122 **	−0.113 *	−0.119 **	−0.168 ***	−0.138 **

Note. * *p* < 0.05, ** *p* < 0.01, *** *p* < 0.001. AAS—Academic Anxiety Scale, Cog-Emot—Cognitive–Emotional Subscale, Acad Mot—Academic Motivation (inversely coded), USDI-Tot—total scale score for all items on the USDI.

**Table 6:** LPA fit results.

Classes	LogLik	AIC	AWE	BIC	CAIC	CLC	KIC	SABIC	ICL	Entropy	Prob_Min	Prob_Max	N_Min	N_Max
1	−3102.63	6221.27	6328.14	6255.70	6263.70	6207.27	6232.27	6230.31	−6255.70	1.000	1.000	1.000	1.000	1.000
2	−2609.14	5244.29	5419.47	5300.25	5313.25	5220.02	5260.29	5258.98	−5350.45	0.866	0.955	0.966	0.470	0.530
3	−2420.75	4877.50	5120.70	4954.98	4972.98	4843.25	4898.50	4897.84	−5027.96	0.877	0.925	0.954	0.165	0.446
4	−2334.52	4715.04	5026.34	4814.04	4837.04	4670.75	4741.04	4741.03	−4917.74	0.855	0.892	0.942	0.088	0.322
5	−2288.67	4633.35	5012.70	4753.87	4781.87	4579.04	4664.35	4664.99	−4885.29	0.849	0.835	0.958	0.066	0.293

**Table 7:** Student profile comparisons for the 4-class solution.

	1 High ER	2 T1—ER	3 T1—Mot	4 T2—ER
N (total = 547)	48	166	176	157
AAS	−0.112	0.036	−0.03	0.03
Lethargy	−0.10	−0.02	−0.05	0.11
Cog-Emot	−0.08	0.01	−0.12	0.15
Acad Mot	−0.31	0.03	−0.02	0.09
GPA ^a^ (N = 396)	3.10	3.18	3.09	2.99
Age ^a^ (N = 512)	22.3	21.6	22.5	21.5
Pct Female ^a^ (N = 539)	66.7%	52.4%	51.4%	46.1%
Child Abuse ^a^ (N = 539)	14.9%	20.7%	16.2%	13.5%
Risky Sexual Behavior ^a^ (N = 531)	0%	1.9%	2.3%	1.3%
Substance Use ^a^ (N = 545)	4.2%	5.5%	4.0%	5.1%
**University Classification ^a^ (N = 518)**				
Community (N = 97)	10.3%	25.8%	34.0%	29.9%
Government (N = 180)	8.9%	29.4%	36.7%	25.0%
Private (N = 241)	8.3%	33.2%	27.4%	31.1%

Note. ^a^: while 547 participants completed the survey items, individual outcome variables (e.g., GPA) were left unanswered by students. For those analyses, pairwise deletion resulted in reduced sample sizes.

## Data Availability

The datasets presented in this article are not readily available because of institutional guarantees for large-scale dissemination. Requests to access the datasets should be directed to the first author.

## Appendix A. Academic Anxiety Scale

Please complete the following items using the four-point scale below.

1 = Not at all typical of me
2 = Somewhat typical of me
3 = Quite typical of me
4 = Very typical of me
  1	I often worry that my best is not as good as expected in school.	1	2	3	4
  2	I tend to put off doing school work because it stresses me.	1	2	3	4
  3	I often worry that I am not doing assignments properly.	1	2	3	4
  4	I am less confident about school than my classmates.	1	2	3	4
  5	I have a sense of dread when I am in my classrooms.	1	2	3	4
  6	I tend to find my instructors intimidating.	1	2	3	4
  7	I spend much of my time at school worrying about what is next.	1	2	3	4
  8	There is something about school that scares me.	1	2	3	4
  9	I’m concerned about what my classmates think about my abilities.	1	2	3	4
  10	I often feel sick when I need to work on a major class assignment.	1	2	3	4
  11	I have a hard time handling school responsibilities.	1	2	3	4
